# Unmediated connection of mental health decline and suicide among medical and nonmedical undergraduates during the pandemic of COVID-19: A cross-sectional comparative study

**DOI:** 10.12669/pjms.38.5.5686

**Published:** 2022

**Authors:** Sadaf Konain Ansari, Sadia Yasir Khan, Farkhanda Jabeen, Areeba Riaz, Ali Hamza Cheema

**Affiliations:** 1 Dr. Sadaf Konain Ansari, MHPE. Medical Education Department Community Medicine Department, M. Islam Medical and Dental College, Gujranwala, Pakistan; 2 Dr. Sadia Yasir Khan, MPH, Community Medicine Department Islamabad Medical and Dental College, Islamabad, Pakistan; 3 Dr. Farkhanda Jabeen, MPH., Community Medicine Department, M. Islam Medical and Dental College, Gujranwala, Pakistan; 4 Dr. Areeba Riaz, MBBS., Community Medicine Department, M. Islam Medical and Dental College, Gujranwala, Pakistan; 5 Dr. Ali Hamza Cheema, MBBS, Pharmacology Department, M. Islam Medical and Dental College, Gujranwala, Pakistan

**Keywords:** Anxiety, Depression, COVID-19 suicide, COVID-19 fear, COVID-19 stress, mental health

## Abstract

**Background and Objectives:**

All medicine and healthcare undergraduates were encountered with terminations and delays of professional examinations. These alterations were on topmost of other tasks the COVID-19 pandemic carried out for instance not roaming, covered faces with masks and specifically segregation. This interruption of normal life was a major cause of mental health disaster and it is no surprise that medicine and healthcare undergraduate has had high rates of psychological effects including hopelessness, stress and suicidal thoughts. This study aimed to investigate the unmediated connection of anxiety and stress related mental health decline and suicide among medical and nonmedical undergraduates during the pandemic of covid-19.

**Methods:**

A multidiscipline online cross-sectional comparative study design was chosen for this study conducted from November 2020 to January 2021 with a pre-validated questionnaire to collect responses from sample size 1290. SPSS- 21 used for descriptive analysis of means, S.D, ANOVA and spearman’s correlations. Forward step-wise model of linear regression applies for true significant bivariate relationship (p<.001)

**Results:**

The result shows that all three cohorts were affected. Among the non-medical cohorts, B-Pharmacy students had the high level (p<.001) of anxiety with suicide ideation response (n=200; 39.2%), along with lowest level of envisions care (19.5%; p<.001) in pandemic. Control and independent variable had a strong negative effects on B-Pharmacy and medical students with p<.000.

**Conclusions:**

This study offered more data that the concerns, anxieties and uncertainties caused by pandemic COVID-19, don’t roll out alone but remain as long-lasting problems demanding ongoing attention.

## INTRODUCTION

COVID-19 pandemic reached Pakistan by March 2020 whereupon WHO confirmed the outbreak a worldwide pandemic.^1^ To hold the virus, Pakistan government broadcasted a national level lockdown which had an exaggerated transformation to people’s living.^2^ The Government’s new approaches entirely changed the group dynamic in people.^3^ The prolonged existence of these actions has had widespread social and psychological inferences for the Pakistani medical and non-medical undergraduates too.^4^ When the pandemic of COVID-19 occurred, it upset the whole world. For all medicine and healthcare undergraduates this meant fast modifications to their educational curriculum, reduced student events, and elimination of them from ward-rotations.^5^ The whole thing hit the suspension switch, but time had stirring on**.** Covid-19 caused a worse mental health decline in all ages particularly undergraduates in Pakistan. Undergraduate’s medicine and healthcare final year students still were likely to start their internship.^6^ Future fourth years medicine and healthcare undergraduates required workplace allocation to finished clinical exposure in their relevant domain of curriculum.^7^ Third and second years still obligatory to get transitioning into pre-clinical sciences and first year were considering onward move to rejoice their first year completion.^8^

All medicine and healthcare undergraduates were encountered with terminations and delays of professional examination. These alterations were on topmost of other tasks the COVID-19 pandemic carried out for instance not roaming, covered faces with masks and specifically segregation.^9^ This interruption of normal life was a major cause of mental health disaster and it is no surprise that all students has had high rates of psychological effects including hopelessness, stress and extremely anxious suicidal thoughts.^10^

In Pakistani social-and-ethnic context youth perspective and their mental health services may not be considered as a thoughtful alarms and may go unobserved. The negligence of such kinds may lead to increased recklessness emotional behavior and resultant suicide.^11^ Therefore the present study explored this unmediated connection of mental health decline and suicide among medical and nonmedical undergraduates during the pandemic of covid-19. And in order to complete the aim of this study a hypothesis was made that there was a relationship between decline mental health wellbeing and suicidal ideation under extreme circumstances.^12^ To date there was no cross-sectional comparative study involving medicine and other healthcare undergraduates. Hence, highlighting this break in literature, COVID-19 psychological responses and other related indicators were compared among undergraduates from different colleges who shared same premises, values and cultural background and had applied similar tactics to fight against COVID-19. A pre-validated questionnaire was used to collect responses.^13^

## METHODS

A multidiscipline online cross-sectional comparative study design was chosen for this study conducted from November 2020 to January 2021.

### Study population and sample size:

The sample consist of all medical undergraduates (4 MBBS sessional years; n=460) from M. Islam Medical and Dental College, Gujranwala; four sessional years of B-Pharm; n=480 from M. Islam college of pharmacy and 4-sessional years of DPT; n=350 students of M. Islam Institute of Rehabilitation and Physiotherapy with aged between 18-to-26-years (mean age=21.74; S.D=2.24).

### Data collection procedure:

After the approval from Institutional Research Ethics Board (IREB letter #: 001/2020) all students were requested to log-in through their personal id allotment to avoid any repetition of responses. Anonymity and confidentiality of undergraduates were secured through specific number generating order, in this way record of each partaker was maintained for further correspondence. Information was collected in two rounds; first round or pilot study was before their final internal assessment exams during the month of November 2020 just to check the accuracy of data collection methods, and second round was collected in January before their professional external assessment exams in February 2021, which was used as empirical results.

### Variables

***1. Data about Participant’s particulars:*** Details regarding their gender, age, and level of sessional years were collected from the survey. Students with medicine batches were classified as Med. cohorts, and others were categorized as Non-medical cohorts. Age and gender is not considered as variable.

***2. Dependent outcome variable:***

***i) Anxiety and suicide ideation:*** In order to assess the pandemic COVID-19 generated sadness, anxiety, and suicide level of participants, *State*-Trait Anxiety Inventory (STAI) was used.^14^ The lower the score means low anxiety (Cronbach alpha of scale was .89), and for COVID-19 related sadness suicide ideas in the past 15 days on Likert scale of 5-point (not at all to very severe). The Coronavirus Reassurance-Seeking Behaviours Scale (CRBS) was used. Static condition of lowered score sadness for more than two weeks was considered as proxy to suicide idea (alpha= .90).^15^

***3. Control variate:***

**a)** Certainty in Pandemic Switch: Certainty in pandemic switch included self-assurance concerning personal, town, and the institutional management in the pandemic condition. The Cronbach alpha was .83.^16^

**b)** Sensitivity of Exposure to COVID-19 risk: The Cronbach value was .80.^17^

**c)** To envision Care: Believed to get support detects perceived help from peers, teachers and families. The Cronbach was .69 of this 5-point Likert scale.^18^

***4. Independent Covariates:***

a. Abundance of Means perceived to fights COVID-19: Sufficient resources of multiple means comprised of; information about COVID-19 situation; currency; approachable healthcare facility; personal protective equipment (PPE); and mental-health support. The Cronbach value was .76.^19^

b. Delivering COVID-19 Facts and figures: Seeking evidence from several sources involved information from: The medical worker, Friends-or-families, and internet-based available data.^20^

### Statistical Analysis:

Descriptive and inferential statistics of mean and standard deviation calculated and applied Kolmogorov-Smirnov and ANOVA- test to examine the variances differences. That was followed by Post-hoc-Tukey HSD adjustments and spearman’s rho correlation to measure the true significant bivariate relationship among participants and all the variables. Thereafter, forward stepwise model used in regression analysis to find the significant effects of variables for multi-groups to address the role of independent covariates in anxiety and suicide ideation among cohorts. Considered P value < .001 was significant.

## RESULTS

### Descriptive and inferential studies:

Total sample size was 1290, out of which 38.1% (N=460; female. n= 282, male. n= 178) enrolled in medicine. Moreover, 37.2% (N= 480; female. n= 345, male. n= 135) in B-Pharmacy. Whereas, 27.1% (N=350; female. n= 200, male. n= 150) in DPT respectively. The total female participants population was 64.1% (female n=827/1290) out of which 61.3% was studying medicine (282/460), 73.0% in B-Pharmacy (345/480) and 57.1% in DPT (200/350) respectively. Furthermore, 43.0% male participants were studying DPT (150/350), 40.0% in medicine (178/460) and very less (28.1%) enrolled in B-Pharmacy (135/480). Gender, age and years of undergraduate level were not considered in variable therefore it was not presented in the results. Table-I.

**Table I:** Demographic properties of different cohorts (N=1290).

Demographic values and tests-items	Cohorts of participants			Mean ( S.D)	ANOVA			Kolmogorov-Smirnov Z test ^a^	
	
	Medical cohort N=460	Non-Medical cohorts			Mean Square Between group, (within group)	F	P value*
	
		B-Pharm N=480	DPT N=350					Statistic	Sig.**
Total Yes; n= 982 (100%)	320 (32.6%)	352 (35.8%)	310 (31.6%)	1.98 (.801)					
Total No; n = 308 (100%)	140 (45.5%)	128 (41.6%)	40 (13.0%)	1.67 (.693)					
Anxiety and suicide ideation; n= 510 (100%)	120 (23.5%)	200 (39.2%)	190 (37.3%)	2.13 (.768)	225.392 (.148)	1526.656	.000	10.919	.000
Certainty in pandemic; n= 499 (100%)	222 (44.5%)	156 (31.3%)	121 (24.2%)	1.79 (.804)	215.363 (.216)	998.518	.000	7.433	.000
Sensitivity of exposure to risk; n= 643(100%)	229 (35.6%)	189 (29.4%)	225 (35.0%)	1.99 (.840)	320.587 (.208)	1540.589	.000	9.073	.000
Envision care; n= 661 (100%)	342 (51.7%)	129 (19.5%)	190 (28.7%)	1.77 (.867)	367.815 (.196)	1875.626	.000	12.020	.000
Abundance of Means perceived to fights; n=720 (100%)	300 (41.7%)	219 (30.4%)	201 (27.9%)	1.86 (.823)	191.070 (.147)	1301.672	.000	5.677	.000
Information from medical worker; n= 649(100%)	302 (46.5%)	236 (36.4%)	111 (17.1%)	1.70 (.742)	266.251 (.140)	1902.684	.000	12.020	.000
Information from internet; n=798 (100%)	300 (37.6%)	289 (36.2%)	209 (26.2%)	1.88 (.790)	208.222 (.103)	2014.340	.000	2.63	.000
Information from families & friends; n=777 (100%)	289 (37.2%)	281 (36.2%)	207 (26.6%)	1.89 (.792)	193.453 (.130)	1490.767	.000	2.801	.000

* p<.001 is significant; a= Lilliefors significance correction;

** grouping variable: total yes is significance.

### Hypothesis Verification-Main analysis:

It was evident from Table-I that among the non-medical cohorts, B-Pharmacy students had the high level (p<.001) of anxiety with suicide ideation response (n=200; 39.2%), along with lowest level of envisions care (believed to get support (19.5%; p<.001) in pandemic. Besides, medical cohort had the strong confidence (44.5%; p<.001) related to pandemic control and personal health. They also had the highest supporting circle (51.7%; p<.001) and information level (46.5%; p<.001). Further, medical cohort had the highest sensitivity of exposure to covid-19 risk (35.6%; p<.001) compared to non-medical cohorts of DPT (35.0%; p<.001) and B-Pharm (29.4%; p<.001) respectively. Therefore, Tukey-test for Post-Hoc analysis was applied to define the exact differences among independent and controlled variable with dependant variable. It was also marked significant in all variable correlation test of spearman’s rho at significant 2-tailed p<.0001 level. These conclude that besides there was a difference in variables’ effects, there was a strong true bivariate relationship in them. The results were presented in Table-II below.

**Table II:** Multiple comparisons Post-hoc analysis and bivariate correlation test. (N=1290).

Tukey HSD								Spearman’s rho	

Variables	Total yes (I)	Total yes (J)	Mean Difference(I-J)	Std. Error	Sig.*	95% confidence interval		N, Correlation coefficient (r)	Sig. (2-tailed)**

						Lower bound	Upper bound		
Abundance of means perceived to fight	Medical cohort	B. Pharmacy	-1.37216*	.02959	.000	-1.4417	-1.3027	720 (.889)	.000
		DPT	-1.93750*	.05930	.000	-2.0768	-.1.7982		
	B. Pharmacy	Medical cohort	1.37216*	.02959	.000	1.3072	1.4417		
		DPT	-.56534*	.05895	.000	-.7038	-.4269		
	DPT	Medical cohort	1.93750*	.05930	.000	1.7982	2.0768		
		B. Pharmacy	.56534*	.05895	.000	.4269	.7038		
Information from internet	Medical cohort	B. Pharmacy	-1.17330*	.02483	.000	-1.2316	-1.1150	798(.921)	.000
		DPT	-1.93750*	.03381	.000	-2.0169	-1.8581		
	B. Pharmacy	Medical cohort	1.17330*	.02483	.000	1.1150	1.2316		
		DPT	-.76420*	.03338	.000	-.8426	-.6858		
	DPT	Medical cohort	1.93750*	.03381	.000	1.8581	2.0169		
		B. Pharmacy	76420*	.03338	.000	.6858	.8426		
Information from families and friends	Medical cohort	B. Pharmacy	-1.19290*	.02782	.000	-1.2582	-1.1276	777(.897)	.000
		DPT	-1.90313*	.04051	.000	-1.9983	-1.8080		
	B. Pharmacy	Medical cohort	1.19290*	.02782	.000	1.1276	1.2582		
		DPT	-.71023*	.04006	.000	-.8043	-.6162		
	DPT	Medical cohort	1.90313*	.04051	.000	1.8080	1.9983		
		B. Pharmacy	.71023*	.04006	.000	.6162	.8043		
Information from medical worker	a	a	a	a	a	a	a	649 (.906)	.000
Envision care	a	a	a	a	a	a	a	661(.889)	.000
Sensitivity of exposure to risk	a	a	a	a	a	a	a	643(.840)	.000
Certainty in pandemic covid-19	a	a	a	a	a	a	a	499 (.804)	.000
Anxiety and suicide ideation	a	a	a	a	a	a	a	510 (.895)	.000

* The mean difference is significant at 0.05 level; a= not applicable;

** correlation is significant at 0.01 level (2-tailed)

**Fig.1:**
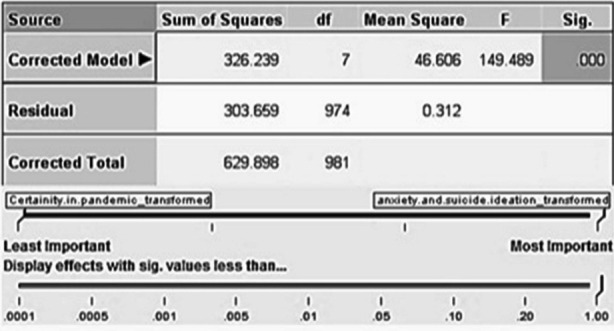
Forward stepwise modelling showing effects of variables on cohorts after linear regression.

**Fig.2:**
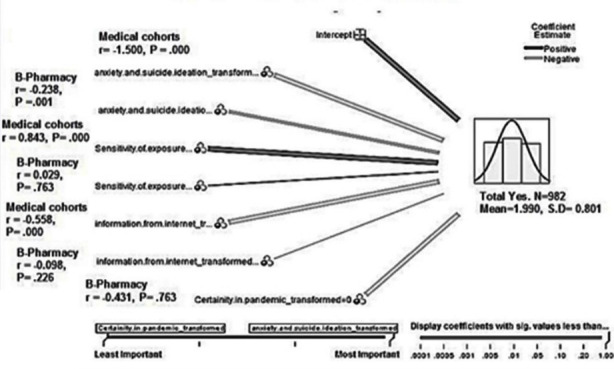
Forward stepwise modelling showing coefficients and significance of variables in cohorts after linear regression.

Additionally, investigating the true nature of association between independent and control variable with outcomes variable, a forward-stepwise model using the information criterion was applied through linear regression analysis. The variables ’effects and coefficients are shown in Fig.1 and 2 with the significant predictor importance of anxiety and suicide ideation during pandemic of COVID-19 in Grapgh-no.1 below.

The regression analysis tells us that control variable; i.e. certainty in pandemic COVID-19 switch and independent variable; i.e. information from internet had a strong negative effects on B-Pharmacy and medical students with p<.000 (Fig.2), whereas, control variable; i.e. sensitivity of exposure to COVID-19 risk had a strong positive effects on Medical cohorts (p=.000) and weak positive effects on B-Pharmacy and DPT students (p=.763) (Fig.2).

## DISCUSSION

The trajectory of decline mental health among students leading to extreme negative emotional behaviour in the form of suicide has been greatly emphasized in the past. This negative emotional behaviour or suicide idea is very impulsive because of unbearable stress, anxiety and depression due to multiple reasons while they were in their studentships. The data used in this study cover entire Islam group of three college population in Gujranwala, and we inspected whether anxiety and suicide ideation had a relationship with decline mental health wellbeing specifically near summative assessment of college and university under extreme circumstances during COVID-19 period. The results verifies the hypothesis that there was an unmediated connections of decline mental health wellbeing with suicidal ideation under strong stress and anxiety (medical cohort; r= -1.500, p=.000), (B-Pharmacy; r=-.0238, p.001).

These findings strongly endorsed the results of another previous study^10^,^12^ This study used forward-stepwise model using information’ criterion transformation affects that control independent variable impact on dependant variables (sadness-anxiety-suicide). The result shows that all three cohort was affected (ANOVA; mean square between group=225.392 and mean square within group =.148 with F= 1526.656, and p=.000) throughout in analysis the anxiety and suicide thoughts to independent variables (abundance of means perceived to fight COVI-19; information from internet and information from friends and families) had the most powerful influence (predictor importance 0.06). That was presented in Graph-1. These findings of present study confirm the findings of another study^21^ This represents that there was no solitary reliable means of information on which students can trust; as misleading information either from internet, friends and families can produce an effects like “echo-chamber” resulting in more anxiety, uncertainty and more stressor in the form of post traumatic experience especially in pandemic time of COVID-19. It is an alarming projection of undergraduates’ mental health decline.^22^

**Graph No.1:**
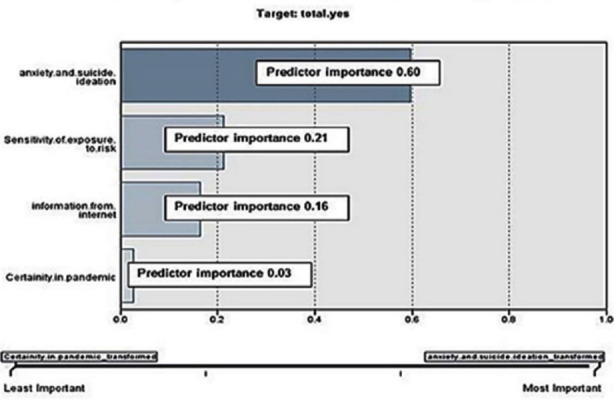
chart showing significant predictor importance in COVID-19.

The sprouting of false or unclear knowledge from any sources is a lot of whistles for tsunami in psychiatric health care system in Pakistan which needs to be reforms and readdress. Regarding comparison of sources of COVID-19 information it was evident that DPT students had less information connections from medical health worker (n=111; 17.1%), internet (n=209; 26.2%), and friends or families (n=207; 26.6%) compared to B-Pharmacy and medical cohort. This represents that despite the fact that they were less informed but they are very anxious (n=190; 37.3%; p<.001)) than medical students (n=120; 23.5%; p<.001) and are also equally affected. These findings of medical and non-medical undergraduates of high education institutions support the outcomes of another study in university students.^23^

Regarding the independent variables effects, it was obvious from outcomes that B-Pharmacy students had very weak envision cares from family; friends; peers; teachers; and medical worker (n=66; 19.5%; r=.889; p=.000). It could be due to the social distancing and online learning from home because of COVID-19 which is another invisible factor in the occurrence of anxiety and sadness resulting into stress-related-wrong-thoughts.^24^

The results of this study emphasis that the risk of undergraduate student (all three cohorts) with suicidal ideation during the pandemic of COVID-19 should not be ignored. Fear is adequate of vulnerability and magnifying the effects of social distancing, isolation and loss of outdoor college-gathering activities with friends and classmates, which ultimately results in by no means-ending anxiety and depression.^25^ The present cross-sectional study recognised that undergraduate’s medical and non-medical students had an increase in stress related anxiety especially at the time of their college level summative assessments in November 2020 and exit professional examination in January 2021, which leads to an unheard depression and if right information and right support was not there it could ends with strong negative emotional behaviour that was; suicide ideations. The present study concludes that medical institutions dealing with healthcare teaching facilities should provide stronger grievance and help concerning mental health of students as pre-emptive methods to slow down the lasting effects of COVID-19 pandemic.

### Limitations of the study:

This study is restricted to undergraduates related to medical field directly or indirectly and not every undergraduate of other subjects of other institutes. Another limitation was the removal of gender, age and study year. These could be possible mediator and could alter impacts of COVID-19 anxiety and suicide in outcomes. Thirdly the exclusion of any pre-existed mental health issue among undergraduates which may reoccurs during the COVID-19 pandemic and produces such negative effects.

## CONCLUSIONS

People connection and support even in the form of virtual setting is the key to fight unpredictable circumstances such as COVID-19. This study offered more data that the concerns, anxieties and uncertainties caused by pandemic COVID-19, don’t roll out alone but remain as long-lasting problems demanding ongoing attention.
